# Influence of distance between residence and health facilities on non-communicable diseases: An assessment over hypertension and diabetes in Bangladesh

**DOI:** 10.1371/journal.pone.0177027

**Published:** 2017-05-18

**Authors:** Raaj Kishore Biswas, Enamul Kabir

**Affiliations:** School of Agricultural, Computational and Environmental Sciences, University of Southern Queensland, Toowoomba, Queensland, Australia; University of West London, UNITED KINGDOM

## Abstract

**Objective:**

This paper reflected on the prevalence of hypertension and diabetes in Bangladesh, which is spreading rapidly in low-income countries. The rationale of constructing more health centers for addressing NCDs was assessed in this paper by determining the relationship between prevalence of NCDs, particularly hypertension and diabetes, and distance to health facilities.

**Methods:**

From BDHS (Bangladesh Health and Demographic Survey) 2011 data set, 7544 samples were analyzed to demonstrate association between Non-communicable diseases (NCD) and distance from respondents’ home to health facilities like hospitals, community clinics, pharmacies or doctors’ chambers, and community facilities like market, post office or cinema hall. Bivariate analysis was conducted between accessibility to health facilities and prevalence of the diseases. The causal relationship between the spatial effects and the prevalence of the diseases were analyzed by applying Generalized Linear Mixed Model (GLMM) was fitted.

**Results:**

Fitting linear mixed effect models, we found that hypertension and diabetes react differently with various spatial effects. Distance from home to hospital had significant effect (*P* < 0.001) on hypertension showing people living further from the facilities or town centers seemed to be less hypertensive, whereas diabetes showed no such affiliation.

**Conclusion:**

Higher prevalence of diabetes (40.9%) over hypertension (26.5%) in people aging 35 or higher, have appeared to have caused the difference, which concluded that each non-communicable disease should be dealt to its own merit for policy making instead considering as a group of diseases.

## Introduction

Non-communicable diseases (NCD) have been the focus of many medical studies for its detrimental effects, both social and economic, in developing countries [1]. One of the unexplored aspect of these diseases is how they are affected by the distances between potential patients’ residence and nearby health facilities, which would help the policy makers to assess the future investments on health infrastructures. Proper allocation of limited resources is essential in a developing country for confronting NCDs. The rationale of constructing more health centers for addressing NCDs was assessed in this paper by determining the relationship between prevalence of NCDs, particularly hypertension and diabetes, and distance to health facilities.

NCDs have actively influenced poverty in developing and under-developed countries, where these chronic diseases lead to continued expenditures trapping poor households in cycles of debt and illness, perpetuating health and economic inequalities. Two-thirds of the deaths in the whole world is attributed by NCDs resulting 36.1 million deaths every year, among which nearly 62% are occurring in the poorest nations [2]. [3] estimated that economic burden of Cardiovascular Diseases totals to around 20% of the state domestic product in Kerala, India and aggregates to USD 30 billion including disability and death in whole India [4]. The cost of medical care along with mortality and morbidity due to diabetes sums to 1.2% of India’s GDP which is 0.4% and 0.6% for UK and Denmark for the year 2007; demonstrating the unfortunate impact in developing countries [5]. The recurrent attack of NCDs bars the individual capacity to contribute in household economy and results in world-wide growth reduction of 0.5% [6]. The social consequence of such diseases are also evident; for example, the stroke survivors and carers have shown to be prone to depression [7, 8]. The economic and social impact was summarized in World Health Organization (WHO) report (2002) stating “in many regions, some of the most formidable enemies of health are joining forces with the allies of poverty to impose a double burden of disease, disability and premature death in many millions of people” [9].

The common impression regarding the spatial effect of health is, as distance between residents and health care providers increases, utilization of health care decreases and vice versa [10, 11]. However, this phenomena should be regarded as one of very many factors for accessibility to health care [12] and even then there exists a psychological comfortableness from the patients’ point of view to have a therapeutic center nearby [13]. Most medical papers investigate on the spatial effect of health facilities in case of determining childhood mortality, maternal health care and other general diseases [14–17]. However, understanding the effect for distance to health facility in case of NCDs like hypertension and diabetes remains a challenge; mainly because of the correlation of such diseases with physical inactivity and high sitting time. [18] showed that physical inactivity causes 6–10% of the major non-communicable diseases like coronary heart disease and type 2 diabetes worldwide. People, in developing nations, living in rural areas or at a distance from the town centers tend to walk or cycle to their destination which prevents NCDs, despite heath facilities are located at a longer distance. The paper investigated this currently unclear phenomenon.

Almost half of the adult diseases in South Asia is attributable to non-communicable diseases [19]. Lack of awareness made Bangladesh an easy victim to hypertension [20]. [21] have calculated that approximately 20% of adult and 40–65% of elderly people suffer from hypertension in Bangladesh and the prevalence is higher in urban areas compared to the rural, due to lifestyle differences [22]. Similar conclusion was drawn in case of diabetes [23], where higher prevalence was found in urban (15.2%, *age adjusted*) areas compared with rural (8.3%) populations and consistent urbanization is deteriorating the status-quo [24, 25]. Besides Nigeria, Bangladesh is the only country in the 10 most populous country’s list which is not among the 10 countries with the highest number of patients with diabetes [26]. However, with the current urbanization and economic growth, NCDs are fast spreading in Bangladesh. Thus, Bangladesh is a perfect sample to evaluate the relation between these two NCDs (Hypertension & Diabetes) and spatial effect of health facilities.

## Methods

### Ethical approval

This article does not contain any studies with human participants performed by any of the authors. We applied a secondary data from DHS (Demographic Health Survey) available online (http://dhsprogram.com/data/). The website provides a list of countries and the link for Bangladesh displays the surveys conducted in Bangladesh. Searching ‘Bangladesh DHS, 2011’ in the DHS website will provide the survey data set.

### Data description

This study used data from DHS (Demographic and Health Surveys), specifically from BDHS (Bangladesh Demographic and Health Surveys) data set of year 2011 [27]. *Measure DHS*+ is a platform where data from developing countries are collected and analyzed on the demographic and health characteristics of population, periodically in an interval of few years [28]. Two different data sets were merged on the basis of cluster ID: one with individual biomarker information where participants’ age were 35 or above and the other one was cluster-wise average distance from respondent’s home to community facilities. Thus we compiled a data set with individual hypertension and diabetes information along with common cluster-wise geographical information demonstrating the average distance to various health facilities. The data set consisted of 7,544 individual level biomarker information and 600 cluster-wise spatial measures.

### Overview of variables

World Health Organization (WHO) employs internationally established and accepted methods for collecting and measuring biomarker measures which is followed by DHS [29]. Individuals aging 18 and above are identified as hypertensive if the average measured blood pressure is raised (SBP ≥ 140 or DBP ≥ 90) or if the adult respondent is actively taking medication for hypertension. If the fasting blood glucose measure is higher than FBG ≥ 126 mg/dl (7.0 mmol/l) then s/he is identified as diabetes patient or if the adult respondent is actively taking medication for elevated blood glucose. It is important to note that some papers never included patients’ medication history while defining the diseases, specifically working on BDHS 2011 data set. The prevalence of the hypertension and diabetes were considered as outcome variables.

Health facilities considered in this study were hospitals, private clinics, satellite clinics, community clinics, NGO clinics, pharmacies, chamber of allopathic/MBBS doctor and homeopathic doctor. Other community centers included weekly market, post office, cinema hall and district headquarter. The distance was measured in kilometers and time in minutes [30]. All the distances were converted into three equiproportional categories namely short, moderate and long distance; except for distance to pharmacies and satellite clinics, which were already dichotomized into two groups by DHS. These are the explanatory variables for the statistical modeling.

### Statistical modeling

Bivariate analysis was conducted between accessibility to health facilities and prevalence of the diseases. And same were applied for the distances to town centers and NCDs. Chi-square test provided the p-values determining the strength of bivariate dependence. To acquire the causal relationship between spatial effects and the prevalence of the diseases, Generalized Linear Mixed Model (GLMM) was fitted. This model is a convenient way to build multivariate distributions for non-normal data that can accommodate some flexibility along with incorporating random effects into the linear predictors [31]. To express the basic model, let ***Y*** be the observed data vector and, conditional on the random effects, ***u***, assume that the elements of ***Y*** are independent and drawn from a distribution in the exponential family; assuming a distribution for ***u*** depending on parameters, ***D*** [32]:

$$\begin{array}{c} f_{\left(y_{i}|u\right)}\left(y|u,\beta ,\phi \right)=exp\left\{\left(y\eta_{i}-c\left(\eta_{i}\right)\right)/a\left(\phi \right)+d\left(y,\phi \right)\right\}u∼f_{u}\left(u|D\right) \end{array}$$ (1)

Here, $\eta_{i}=x_{i}^{\prime }\beta +z_{i}^{\prime }u$, with $x_{i}^{\prime }$ represents *i*th row of the fixed effect ***X*** and $z_{i}^{\prime }$ is the same for random effect ***Z***. The cluster effect was considered as random effect in this paper. The *R*—*package* *glmer*(*lme*4) was applied for fitting GLMM. All computations were conducted in *R* (version 3.2.3).

## Results

### Bivariate analysis

Table 1 displayed bivariate relationship for both hypertension and diabetes with various health features including availability of doctors and medicines in the locality. It was followed by distance to different community facilities from respondent’s residence.

**Table 1:** Association between health characteristics and the prevalence of hypertension & diabetes.

Variable	Hypertension Status			Diabetes Status		
	No hypertension (%)	Hypertension (%)	P-value	Non-diabetes (%)	Diabetes (%)	P-value
**Blood pressure ever checked**						
Yes	3862 (68.9%)	1742 (31.1%)	<0.001			
No	1681 (86.6)	259 (13.4%)				
**Takes Medicine for Diabetes**						
Yes				4131 (100%)	2582 (89.4%)	<0.001
No				0 (0%)	307 (10.6%)	
**Presence of allopathic/MBBS doctors in the village**						
Yes	2136 (71%)	872 (29%)	<0.001	1755 (58.3%)	1253 (41.7%)	0.284
No	3407 (75.1%)	1129 (24.9%)		2704 (59.6%)	1832 (40.4%)	
**Number of allopathic/MBBS doctors in the village**						
One	521 (71.8%)	205 (28.2%)	0.341	446 (61.4%)	280 (38.6%)	<0.001
Two-five	1283 (71.4%)	514 (28.6%)		1050 (58.4%)	747 (41.6%)	
Five+	302 (67.7%)	144 (32.3%)		248 (55.6%)	198 (44.4%)	
No Information	30 (76.9%)	09 (23.1%)		11 (28.2%)	28 (71.8%)	
**Presence of pharmacies in the village**						
Yes	3694 (72.6%)	1394 (27.4%)	0.014	3030 (59.6%)	2058 (40.4%)	0.268
No	1849 (75.3%)	607 (24.7%)		1429 (58.2%)	1027 (41.8%)	
**Number of pharmacies in the village**						
One	473 (75.8%)	151 (24.2%)	0.082	405 (64.9%)	219 (35.1%)	0.002
Two-five	2336 (72.6%)	880 (27.4%)		1862 (57.9%)	1354 (42.1%)	
Five+	885 (70.9%)	363 (29.1%)		763 (61.1%)	485 (38.9%)	
**Weekly Market**						
Short	1883 (75.5%)	610 (24.5%)	0.027	1540 (61.8%)	953 (38.2%)	0.035
Moderate	802 (74.7%)	271 (25.3%)		641 (59.7%)	432 (40.3%)	
Long	1179 (78.8%)	318 (21.2%)		863 (57.6%)	634 (42.4%)	
**Post Office**						
Short	2818 (71.3%)	1135 (28.7%)	<0.001	2368 (59.9%)	1585 (40.1%)	0.151
Moderate	1203 (74.3%)	419 (25.8%)		963 (59.4%)	659 (40.6%)	
Long	1522 (77.3%)	447 (22.7%)		1128 (57.3%)	841 (42.7%)	
**Cinema Hall**						
Short	1845 (69.6%)	807 (30.4%)	<0.001	1587 (59.8%)	1065 (40.2%)	0.088
Moderate	1903 (74.8%)	642 (25.2%)		1460 (57.4%)	1085 (42.6%)	
Long	1795 976.5%)	552 (23.5%)		1412 (60.2%)	935 (39.8%)	
**Time to district headquarter**						
Short	1429 (74%)	502 (26%)	0.008	1125 (58.3%)	806 (41.7%)	0.083
Moderate	1511 (77.4%)	441 (22.6%)		1187 (60.8%)	765 (39.2%)	
Long	924 (18.3%)	256 (21.7%)		732 (62%)	448 (38%)	

Significant association was found between hypertension and people who checked their blood pressure (BP) previously, displayed in Table 1. Among the respondents whose BP was never checked, 13.4% seemed to have hypertension. Similarly 10.6% respondents who did not take any medicine were tested to have diabetes. Significant association were detected between hypertension prevalence and presence of doctors (*P value* < 0.001) and pharmacies in the locality (*P value* 0.014). Existence of diabetes did not show significance with these two factors; however, diabetes had an association with number of doctors in the village (*P value* < 0.001) and frequency of pharmacies in the locality (*P value* 0.002).

### Mixed model analysis

In Table 1, bivariate analysis had been performed to examine the nature of association between the distance characteristics and the current status of diabetes and hypertension. Numerous associations were found to be significant; however, bivariate association between two variables does not necessarily imply a significant causal relationship between them. Therefore, a multivariate approach was applied to determine which distances best explain and predict the prevalence of diabetes and hypertensions, showed in Tables 2 and 3.

**Table 2:** Mixed model fitted for hypertension over distance to community facilities.

Variable		Odds (C.I.)	P-value
Model 1: Random effect (cluster) variance = 1.187			
**Hospital** (Ref: short distance)	Moderate	0.91 (0.77 ∼ 1.08)	0.277
	Long	0.74 (0.62 ∼ 0.89)	<0.001 **
**Private Clinic** (Ref: short distance)	Moderate	0.73 (0.62 ∼ 0.86)	<0.001 **
	Long	0.70 (0.59 ∼ 0.84)	<0.001 **
**Satellite Clinics** (Ref: Inside village)	Outside village	0.92 (0.76 ∼ 1.13)	0.408
Model 2: Random effect (cluster) variance = 1.174			
**Community Clinic** (Ref: short distance)	Moderate	0.93 (0.75 ∼ 1.16)	0.516
	Long	0.94 (0.78 ∼ 1.14)	0.526
**NGO Clinic** (Ref: short distance)	Moderate	0.85 (0.69 ∼ 1.03)	0.101
	Long	0.84 (0.68 ∼ 1.04)	0.108
**Pharmacy** (Ref: close distance)	Far	1.04 (0.88 ∼ 1.24)	0.654
Model 3: Random effect (cluster) variance = 1.204			
**Allopathic/MBBS Doctors** (Ref: short distance)	Moderate	0.89 (0.75 ∼ 1.05)	0.156
	Long	0.81 (0.69 ∼ 0.95)	0.009 **
**Homeopathic Doctors** (Ref: short distance)	Moderate	0.95 (0.81 ∼ 1.11)	0.493
	Long	0.79 (0.67 ∼ 0.95)	0.009 **
Model 4: Random effect (cluster) variance = 1.174			
**Weekly Market** (Ref: short distance)	Moderate	1.07 (0.86 ∼ 1.33)	0.543
	Long	0.89 (0.73 ∼ 1.09)	0.254
**Post Office** (Ref: short distance)	Moderate	1.01 (0.82 ∼ 1.24)	0.932
	Long	0.84 (0.68 ∼ 1.02)	0.082
**Cinema Hall** (Ref: short distance)	Moderate	1.18 (0.94 ∼ 1.49)	0.162
	Long	1.08 (0.85 ∼ 1.37)	0.514

** level of significance at 1%

**Table 3:** Mixed model fitted for diabetes over distance to community facilities.

Variable		Odds (C.I.)	P-value
Model 1: Random effect (cluster) variance = 9.650			
**Hospital** (Ref: short distance)	Moderate	0.91 (0.63 ∼ 1.31)	0.601
	Long	0.69 (0.47 ∼ 1.02)	0.064
**Private Clinic** (Ref: short distance)	Moderate	1.04 (0.73 ∼ 1.49)	0.810
	Long	0.87 (0.59 ∼ 1.27)	0.467
**Satellite Clinics** (Ref: Inside village)	Outside village	0.72 (0.46 ∼ 1.12)	0.149
Model 2: Random effect (cluster) variance = 12.416			
**Community Clinic** (Ref: short distance)	Moderate	0.84 (0.51 ∼ 1.39)	0.502
	Long	1.05 (0.68 ∼ 1.61)	0.823
**NGO Clinic** (Ref: short distance)	Moderate	1.00 (0.64 ∼ 1.59)	0.984
	Long	0.95 (0.59 ∼ 1.53)	0.831
**Pharmacy** (Ref: close distance)	Far	1.35 (0.91 ∼ 1.99)	0.138
Model 3: Random effect (cluster) variance = 9.088			
**Allopathic/MBBS Doctors** (Ref: short distance)	Moderate	1.01 (0.71 ∼ 1.43)	0.950
	Long	0.88 (0.63 ∼ 1.24)	0.472
**Homeopathic Doctors** (Ref: short distance)	Moderate	1.20 (0.86 ∼ 1.67)	0.278
	Long	1.07 (0.75 ∼ 1.53)	0.699
Model 4: Random effect (cluster) variance = 11.787			
**Weekly Market** (Ref: short distance)	Moderate	1.11 (0.68 ∼ 1.81)	0.675
	Long	1.23 (0.79 ∼ 1.90)	0.355
**Post Office** (Ref: short distance)	Moderate	1.15 (0.72 ∼ 1.83)	0.566
	Long	1.11 (0.71 ∼ 1.74)	0.636
**Cinema Hall** (Ref: short distance)	Moderate	1.99 (1.18 ∼ 3.35)	0.009 **
	Long	1.76 (1.04 ∼ 2.98)	0.035 *

* level of significance at 5%,

** level of significance at 1%

The mixed effect models were applied taking clusters as random variable. Hypertension was fitted with spatial effects of health facilities followed by community facilities, shown in Table 2. In every distance variable, the shortest scale was taken as the reference category. Four separate models were fitted to attain maximum optimization and nullify over fitting the models with too many parameters. The random effects showed significant variances (Table 2). Generalized linear model without the random variables showed different results, rationalizing the application of random effects.

Table 3 demonstrated the mixed effect models for diabetes with distance characteristics. Four models were fitted for this NCD too.

Distance to some health facilities (Hospital, private clinic, doctors’ chamber) showed significant effect over hypertension, displayed in Table 2. Interestingly, all the health facility spatial effects, both significant and insignificant, demonstrated unconventional result. For example, respondents were 26% less likely to be hypertensive if hospitals were at a long distance compared to the short distances. Similar effects were concluded for distance from respondents home to private clinics with hypertension was 30% less likely to sustain in people living further away from clinics. The proportion was 19% and 21% with similar interpretations for MBBS and Homeopathic doctors respectively. However, distance to community facilities like market, post office or cinema hall did not pose significant impact on hypertension. As for Table 3, the random effect variance was higher in the GLMMs for diabetes compared to hypertension. The cluster effect was more prominent on diabetes. No impact of geographical distances on prevalence of diabetes were found. Contrasting to hypertension (in Table 2), prevalence of diabetes actually increased as distance to some health facilities increased, however the effects were not significant. Interestingly, distance to cinema hall showed some significance.

## Discussion

The objective of this paper was to determine the relationship between the prevalence of NCDs and accessibility to health care, particularity distance to health facilities from home. Both bivariate analysis and mixed model approach showed that this relationship varies upon diseases; and accessibility to health infrastructure may not be applicable to challenge all NCDs. This study was limited by average cluster distance where we could only attain an average distance to health facility from a cluster, not every household. However, we had enough evidence to suggest that the spatial effect of hypertension and diabetes are different and it appeared to depend on prevalence of the particular disease in the adjacent locality.

There remains many people who have not been checked for their NCDs in Bangladesh. 13.4% (n = 259) respondents who never checked their blood pressure, had been tested to have hypertension. Considering hypertension does not show any eminent symptoms [33] or it could be masked [34], presence of medical check-up is necessary for early detection. This phenomenon was demonstrated in Table 1 where presence of qualified (MBBS) doctors and pharmacies showed significant (*P value* < 0.001) association with hypertension. Among the diabetes patients, 10.6% did not take any medicine to control blood glucose. This lead to an intuitive conclusion that lack of health facilities could be responsible for diabetes patients’ insufficient medicine intake, which was confirmed in the bivariate analysis of Table 1. Significant association between prevalence of diabetes and number of available facilities like number of qualified (MBBS) doctors (*P value* < 0.001) and number of pharmacies (*P value* 0.002) was detected. The second portion of Table 1 gave contrasting views between hypertension and diabetes. Prevalence of hypertension seemed to have significant association with distance from home of respondents to town center represented by weekly market, post office, cinema hall, and district headquarter; whereas such spatial characteristics had no association with diabetes patients’ prevalence.

To further investigate the contrasting association of prevalence and distance between the two NCDs, we fitted both cases in mixed effect model. Distance to hospital, private clinic and satellite clinic from home were found to have significant effect (*P value* < 0.001) on prevalence of hypertension. However, that effect showed a negative association with prevalence and distance; that is as distance from home to heath center increased, the lesser prevalence of hypertension were observed. The unusual phenomenon could be explained from Table 1 where people living further from town centers showed lesser tendency of having hypertension. Table 1 showed percentages of people living further from weekly market had lesser prevalence of hypertension (short = 24.5%, moderate = 25.3% & long = 21.2%). Physical activity actively prevents the occurrence of hypertension by keeping the BP at a tolerable rate [35, 36]. Hence people living further from the town centers have to walk or travel more and the transport system in remote locations of Bangladesh is not ready-available [37], forcing the people to walk or cycle, thus they remain physical active. Furthermore, people in rural areas generally depend on manual labors like farming [38], which helps them to maintain BP in spite of living at a distance from health facilities. The prevalence of diabetes and distance to any health facility had no causal relationship, displayed in Table 3. Two possible reasons could be finalized: either the random effect (cluster) variance was too high to detect any significance or the over-prevalence of diabetes in Bangladesh. The cluster effect were quite high in all four models (more than 9.0), which was more than any covariate variance. On the other hand, diabetes is detected as one of the prominent health threats in Bangladesh prevailing both in urban and rural areas [39] with the second largest number of adults with diabetes (5.1 million adults, 6.31%) in South Asia [40]. Both reasoning concluded that the current magnitude of diabetes prevalence was too high to associate with any spatial characteristics. Among the 7544 biomarker respondents aging 35 or higher, 26.5% (*n* = 2001) appeared to be hypertensive whereas 40.9% (*n* = 3085) were diabetes patients; thus explaining the contrast between the two NCDs.

Health policy makers should not consider the NCDs as a group of diseases, rather individual merit of each disease should be taken into concern. We showed that prevalence of a specific disease had correlation with accessibility to heath care specially the distance between home and health facilities. In terms of allocating budgets, it is suggested that awareness for physical exercise or fitness training should be prioritized as well as building new health infrastructures. Creating awareness is more important for diseases like hypertension which have not spread in a pandemic order in Bangladesh unlike diabetes. Diabetes requires more specilaized hospitals and doctors to control the over prevalence. At present, Bangladesh does not have a community-based public health program for NCDs [41]. Countries like Bangladesh need to make disease-wise policy to properly address the NCDs like diabetes and hypertension, two life-threatening diseases with harmful future prospects [42].

## Conclusion

The non-communicable diseases are spreading rapidly all over the world. Fast urbanization and change in lifestyle has become the Achilles heel for developing countries as they have to face the heavy onslaught of NCDs. With the limited economy, it is difficult for them to make multiple health schemes simultaneously. This paper evaluated the scenario of hypertension and diabetes in Bangladesh to assist the policy makers find focus in tackling NCDs. The results suggested that prevalence of hypertension and diabetes in Bangladesh are not same and availability of health facilities reacted differently with their current prevalence. People living further from the town center, mostly in remote rural areas, were not hypertensive and constructing more health infrastructure will not benefit them; rather awareness to remain fit (maintaining BMI) is more important. However, with higher prevalence, diabetes had spread all around the urban and rural regions, which indicated more cure measures are required for addressing it. Instead of confronting NCDs as a group of diseases, it is important to treat each of them to their individual merit in a particular geographical location.
